# Occupational health: a world of false promises

**DOI:** 10.1186/s12940-018-0422-x

**Published:** 2018-11-21

**Authors:** Joseph LaDou, Leslie London, Andrew Watterson

**Affiliations:** 1 Emeritus Clinical Professor, Division of Occupational and Environmental Medicine, University of California School of Medicine, San Francisco, USA; 2 School of Public Health and Family Medicine, University of Cape Town, Cape Town, South Africa; 3 Occupational and Environmental Health Research Group, Stirling, UK

**Keywords:** Human rights, ILO decent work, ILO conventions, Occupational health, Occupational safety, UN funding, WHO program of work

## Abstract

The response of the World Health Organization (WHO) to the Ebola outbreak in West Africa in 2015 demonstrated that the global health system is unprepared to address what should be its primary mission, control of disease epidemics while protecting health workers. Critics blamed WHO politics and its rigid culture for the poor response to the epidemic. We find that United Nations agencies, WHO and the International Labor Organization (ILO), are faced with the global problem of inadequate worker protections and a growing crisis in occupational health. The WHO and ILO are given monumental tasks but only trivial budgets, and funding trends show UN agency dependence on private donations which are far larger than funds contributed by member states. The WHO and ILO have limited capacity to make the necessary changes occupational health and safety demand. The UN could strengthen the national and global civil society voice in WHO and ILO structures, and by keeping conflict of interest out of policy decisions, ensure greater freedom to operate without interference.

The world’s workforce sustains at least 370 million injuries every year, a figure that would be much higher if reliable reporting existed. Occupational illnesses attributed to hazardous exposures or heavy workloads may be as numerous as occupational injuries [1]. Very few workers worldwide have access to occupational health services that provide for prevention of occupational risks, health surveillance, training in safe working methods, first aid, and consulting with employers on occupational health and safety. Yet access to occupational health services is a right recognized by the United Nations whose absence should be framed as a violation of the right to health [2, 3].

There are nearly 3 million workers known to die each year from occupational injuries and diseases. Diseases related to work cause the vast majority of deaths among workers. Occupational cancer is responsible for almost a third of all work-related deaths. More than one million workers die each year due to exposure to hazardous substances. The overall worker death rate is steadily increasing. The vast majority of these deaths are occurring in the poorest of countries with the least legal protections for its workers, yet they are avoidable and preventable [4].

The global epidemic of occupational injury and disease is not new. It is inherent in the nature of industrial development that poorer countries are left with far more than their share of hazardous production and unsafe work environments. Growing economic competition has led some countries to compete, not only in the quality and productivity of work, but also in minimizing the costs of labor by paying less than reasonable minimum wages. Poverty and poor social conditions too often have serious adverse impacts on workers’ health. Moreover, standards such as those for occupational health and safety may be set far below those accepted in UN International Conventions. While international standards appear to obligate employers to provide occupational health and safety procedures, and to pay for occupational injury and disease, inadequate prevention, absence of worker protections, and a failure to provide compensation make a mockery of these standards.

## Global working conditions

More than 1.4 billion people, almost four out of five workers in developing countries, work in hazardous settings or occupations. The number of workers in vulnerable employment increases by around 11 million each year [5]. Developing countries seldom have enforceable occupational and environmental regulations, and even in many developed countries, populist governments are moving away from workplace regulation and enforcement. Occupational health and safety laws cover only about 10% of workers in developing countries. These laws omit many major hazardous industries and occupations. Progress in bringing occupational health to the industrializing countries is painfully slow. In the poorest countries, there has been no progress at all.

Occupational health should have high priority on the international development agenda because occupational injuries and diseases have a serious impact on the economy of all countries. Occupational injuries cause permanent disabilities and economic losses amounting to 4–6% of national incomes, costs to developing countries in excess of $10 trillion [6]. Similar costs are anticipated from occupational diseases although studies are only now being considered [7]. These preventable injuries and diseases also have profound impacts on the work productivity, income, and social well-being of workers and their families.

Often ignored is the reality that a single occupational injury or illness can tip an entire family into poverty. The UN Universal Sustainable Development Goals (USDG) emphasise the need to prevent catastrophic illness from tipping families into poverty but has a blind spot in that it does not recognise occupational causes of such catastrophic illness and injury--if it did, it would emphasise prevention of workplace illness and injury. USDG calls upon developed countries to assist the development process in developing countries, particularly the least developed countries and to deliver on their long-standing pledges to commit 0.7% of their Gross National Income (GNI) to official development assistance programs, again without explicit mention of occupational health and safety [8].

The workers most vulnerable to workplace injury and disease are those with the least secure employment, low incomes, long hours, virtually no unionization, and inadequate diets, housing, transport, and access to broader health care or social security nets. Migrant workers, seasonal workers, indigenous workers, women, and child workers are the most likely to be exposed to hazardous and toxic work, financial and sexual exploitation, environmental pollution, systems of workplace organization injurious to heath, and social deprivation. In developing countries, workers are threatened in many ways with little government protection, from simply losing their jobs if they speak up, to losing their lives. There is remarkably little objection to deplorable working conditions anywhere in the world. Unions provide a protective effect on workers’ safety. Anti-union legislation increasingly advanced in populist governments has a deleterious effect on occupational health [9].

## The global response

Many countries and organizations have attempted to deal with the problem of worker protections and occupational health. The efforts are seldom sustained long enough to make any real difference. The United Nations (UN) budget is just large enough to create a public relations effort suggesting that the problem is being addressed, which it is not. This largely paper program provides an opportunity for most countries to simply agree to the principles, and to essentially ignore the problem.

Academic institutions use the developing world as a place for clinical and research training, again with little or no effect on worker protections and occupational health. Global Health is in danger of becoming a funding stream that generates ‘global health’ programs and institutes that may do research and training in poor countries, but whose esssential purpose is to capture a share of the funder market for their institutions, staff and students.

One example of the scope of the problem--and the inadequate international response--is seen in worker migration. The world’s largest population migration is taking place at this time – one in seven of the world’s people are on the move. In 2003, the UN adopted the International Convention on the Protection of the Rights of All Migrant Workers and Members of Their Families to guarantee equality of treatment and the same working conditions for migrants and nationals. Not one single migrant-receiving country in Western Europe or North America has ratified the Convention.

Many thousands of migrant workers on construction sites in Qatar, including those building stadiums for the 2022 World Cup, are subjected to potentially life-threatening heat and humidity. The campaign group Human Rights Watch (HRW) claims that the Qatar authorities have refused to provide information to the public on hundreds of workers who die every year, or to properly investigate the deaths. Working conditions in the region’s fierce climate places millions of workers at risk, including those in the other Gulf Cooperation Council (GCC) countries–Bahrain, Oman, Kuwait, Saudi Arabia and the United Arab Emirates [10].

## International agencies

Most countries defer to the United Nations guidance of international occupational health. The UN’s international agencies have had a very limited success in bringing occupational health to the industrializing countries. The lack of proper World Health Organization (WHO) and International Labor Organization (ILO) funding severely impedes the development of international occupational health.

There are 194 UN member states that agree to support the activities of the WHO and the ILO. The WHO structure is designed to limit the power of any one member state to influence policy or direction. However, not all UN agencies provide democratic channels for global governance. For example, the Rotterdam Convention, which should include all forms of asbestos and many pesticides in the schedule of highly hazardous chemicals, allows single countries to block rulemaking, which powerful countries use to protect their hazardous industries. Moreover, all member states are meant to contribute a proportion of the core WHO and ILO budgets based on their wealth and population size. Member states are supposed to provide the support regardless of agency priorities or performance. However, member states increasingly attempt to influence the actions of WHO and ILO by threatening to quit membership, or more stealthily by proposing changes to the budget.

The WHO International Agency for Research on Cancer (IARC) provides unbiased evaluation of products introduced commercially by industry. Yet even IARC, with its highly regarded reputation, has witnessed an infiltration by industry forces in recent years. After the breakup of the Soviet Union, Russia stopped paying annual dues to IARC. Some years later, Russia resumed payments, followed by pressure to get IARC to collaborate with a discredited Russian institute to conduct epidemiologic research on Russian miners, relying on gravimetric analysis of asbestos rather than fiber counts. IARC later participated in a Kiev conference, organized to promote the continued use of asbestos, and the publication of a paper (co-authored by IARC staff) with several industry-propaganda assertions [11].

The WHO and ILO may receive voluntary contributions from most any source, including corporations, other organizations such as trade associations, and individuals. The reliance on voluntary contributions to the operation of the WHO and ILO has increased dramatically over the past two decades. Voluntary contributions now comprise about 80% of the WHO’s overall budget. In 2017 the ILO received voluntary funding from donors of $375 million, about half its total funding [12]. Moreover, member states may use their contributions to act in the interests of their country’s corporations. There is no public accounting of the inherent conflicts of interest in this funding arrangement.

Voluntary contributions are characteristically designated for specific purposes proposed by the donor. Which leaves the WHO and ILO open to member state and corporate mischief, influence, and outright control [13, 14]. The benefits to the tobacco industry, asbestos and other mining and manufacturing industries are achieved with rare public reporting. It is quite likely that the trivial WHO and ILO funding and human resource allocated to support occupational health and safety are the result of donor influence and control of governance.

- World Health Organization

The WHO is responsible for the technical aspects of occupational health and safety, the promotion of medical services and hygienic standards. The WHO global policy on occupational health addresses occupational health through a network of unfunded Collaborating Centers. The concept is consistent with overall WHO policy of institutional innovation, broadly defined as ‘network governance’, by which collective action is achieved through interconnected institutions spanning government, business and civil society [15].

Many Collaborating Centers are major governmental and academic institutions. The National Institute for Occupational Safety and Health (NIOSH) in the United States defines it primary international effort as one of participation in the writing of WHO documents as a Collaborating Center. The most recent example of this collaboration is a WHO document about preventing disease through a healthier and safer workplace [16]. In the widely circulated document there is not one mention of trade and inequity as a cause of the global burden of disease associated with work; and not one mention of human rights. There appears to be no formal editing process at WHO before contributions go into print.

The problem is that such participation with WHO limits the interest member states have in pursuing further avenues of assistance to developing countries. The prominent position taken by Finland in occupational health and safety is widely respected, but it may have been used by some countries as a reason for inaction instead of an exemplar of what can and should be done globally. This provides most member states with an excuse to turn their attention to other issues.

The WHO produces a blizzard of paperwork that states virtually every possible goal of an international program of occupational safety and health, none with any measured effect. The WHO plan to protect workers and prevent illness and injury is periodically stated in work programs. The 12th General Program of Work 2014–2019 proposed to establish “health protection at all workplaces, to decrease inequities in workers’ health between and within countries, ensure access of all workers to preventive health services and link occupational health to primary health care, improve the knowledge base for action on protecting and promoting the health of workers, and to stimulate incorporation of actions on workers’ health into other policies, such as sustainable development, poverty reduction, trade liberalization, environmental protection, and employment” [17].

The 12th General Program of Work does not appear to recognize what the WHO Commission on Social Determinants of Health stated so clearly—that “the conditions in which people live and die are, in turn, shaped by political, social, and economic forces” and that “the unequal distribution of health-damaging experiences is not in any sense a ‘natural’ phenomenon but is the result of a toxic combination of poor social policies and programs, unfair economic arrangements, and bad politics” [18].

The 13th Global Program of Work 2019–2022 is currently in draft and risks continuing this pretense. Although the new program will add more goals, e.g., “Protect against climate and environmental change, support national health authorities to focus on green health facilities; substantially reduce the number of deaths and illnesses from hazardous chemicals and air, water and soil pollution and contamination, and improve water and sanitation, and energy” [19], the likelihood of meaningful changes emerging from this program are slim.

There is a growing problem of credibility with the WHO, a problem exploited by the private sector to shift authority for key decision-making in occupational health and safety away from the WHO to other UN agencies and to the private sector itself. Lack of trust in the management of the WHO could partly explain why funding agencies are increasingly becoming directly involved in defining how and on what their money should be spent. In all too many cases, organizations and member states use the WHO imprimatur to burnish their reputations as contributors to an international effort, but offer little in the way of participation.

Moreover, the selection of WHO Collaborating Centers is arbitrary, and fails to protect the organization from opportunistic participants. This makes WHO Collaborating Centers soft targets for industry manipulation. As an example, consultants affiliated with Philip Morris (PM), the world’s leading tobacco corporation, established close links with the Chulabhorn Research Institute (CRI) in Bangkok. Over a period of many years they were able to influence scientific research and debate around tobacco and health, to link with academic institutions, and to develop relations with key officials and local scientists so as to advance the interests of PM within Thailand and across Asia. During this period, the CRI assumed international significance with its designation as a WHO Collaborating Center [20].

A decade later, PM announced its support for the establishment of a new entity - the Foundation for a Smoke-Free World, and to support the Foundation by contributing approximately $80 million annually over the next 12 years. UN Guidelines now state that governments should limit interactions with the tobacco industry and avoid partnership, but no attempt was ever made to explain the activities of the CRI-WHO Collaborating Center [21]. Moreover, the first CEO of the Foundation for a Smoke-Free World, a supposedly-independent research foundation established by PM to promote its new range of smokeless-tobacco products is Derek Yach. Yach is a former senior WHO official, who drove the WHO’s program of work on the Framework Convention on Tobacco Control and his fronting of the Foundation reflects a very high level of capture of key policy makers by corporate interests.

- International Labor Organization

The ILO is the only UN tripartite organization consisting of government, employer, and worker representatives designated ‘to set labor standards, develop policies and devise programs promoting decent work for all women and men’ [22]. The ILO adopts two kinds of standards: Conventions and Recommendations. Only Conventions can be ratified and thus become legally binding on member states.

The ILO Conventions guide all countries in the promotion of workplace safety and in managing occupational health and safety programs. Ratification by member countries is entirely voluntary. No sanctions are provided against member countries that do not ratify conventions, and there is usually no time limit set for ratification. Moreover, even if a country has ratified a convention, the ILO cannot enforce compliance.

Ratifications are made by a disappointingly small percentage of ILO member states. The Convention that seeks to promote a safe and healthy working environment has received only 43 ratifications. Conventions directed at managing occupational health and safety programs have only 33 ratifications, Conventions for safety and health in construction, 31 ratifications, and safety and health in agriculture, a mere 16 ratifications.

The ILO Tripartite Declaration of Principles Concerning Multinational Enterprises and Social Policy (MNE Declaration) asserts that the ILO provides direct guidance to enterprises on social policy and inclusive, responsible, and sustainable workplace practices. The ILO states that the MNE Declaration is the only global instrument adopted by governments, employers and workers from around the world [23]. This is yet another example of sweeping assertions that something is being done but without any measure of reality.

The ILO Decent Work program has been a means of generating interest in and some action on worker protections in industrializing countries. The ILO is a major source of information for government, employers and workers. The ILO Labor administration assists constituents in promoting Decent Work through the strengthening of labor administration machinery, including labor inspection [24]. Yet employers and workers are also calling for better resources for Ministries of Labor and inspectorates to make Decent Work a reality [25].

The number of workplaces subject to inspection dwarfs the resources available to inspect them, leading to a situation in which workers are unprotected, violators operate with impunity, and unfair competition for compliant businesses pervades [26]. ILO’s strategic compliance model provides labor inspectorates with a methodology to achieve compliance outcomes in light of limited resources [27]. Independent assessment of such programs will be needed to evaluate their full effectiveness.

The ILO has a number of highly controversial projects with industry. The stated purpose of ILO technical cooperation with industry is the implementation of the Decent Work agenda at a national level [28]. None of these industry-friendly projects is more controversial than the ILO acceptance of funds from the tobacco industry, presumably to help in the control of child labor in Africa. The ILO receives funding from the Eliminating Child Labor in Tobacco Growing Foundation (ECLT), a non-profit foundation operating under the supervision of the Swiss government, but funded by tobacco companies. To date, the total amount of funding received by ILO from the ECLT is $5,332,835 [29]. Notably, critics have argued that industry-funded child labor projects such as ECLT are primarily used to enhance corporate reputations and conceal the fact that the economic benefit of ongoing use of child labor by large tobacco corporations outweighs by more than 16-fold the amount of money budgeted for ECLT [30–32].

A majority of the ILO member states want the organization to end its financial ties with the tobacco industry. However, employer groups and a few countries, mostly in the African region, continue to defend the industry program. The ILO partnership with the tobacco industry has no record of achievement, and fails to address the root causes of child labor. Ending child labor in tobacco-growing countries can be achieved without accepting money from the tobacco industry [33].

## Current status

Nearly half of the working-age population around the world is unemployed, inactive, or underemployed [34]. The global workforce is facing increasing unemployment as transnational corporations build state-of-the-art production facilities where machines replace workers. Low-skilled workers with limited education cannot compete with the cost efficiency, quality control, and speed of delivery achieved by automated manufacturing [35]. Moreover, automation is also occurring in the largest global employment sectors - agriculture, manufacturing, and services.

In developing countries, nearly 780 million workers, about one in three, live in moderate to extreme poverty. These workers are less likely to have secure jobs with regular incomes and access to social protection. They are in need of a serious effort to assure worker protections and occupational health. Yet these impoverished workers are in no position to make demands, and if they do make demands, they are not likely to be received. There is a glaring weakness of organized labor around the world, and its absence contributes to the overall problem of worker protections and occupational health. However, there are a number of small but global and regional trade unions and NGOs, with even less funding and fewer staff than much better resourced international agencies, that have been able to reach workers with information, advice and support on occupational health and safety matters and contribute to improving working conditions.

Developing countries are far behind industrialized countries in the development of workers’ compensation programs. In many countries of Asia, Latin America, and Africa, only a small fraction of the workforce is covered by workers’ compensation programs. In countries as large as Egypt, India, Pakistan, and Bangladesh, fewer than 10% of workers are covered by workers’ compensation. Developing countries, including China, seldom reach a level of 20% coverage. In many developing countries, workers’ compensation is little more than a paper program where the government works in concert with industry to minimize the provision and the costs of benefits.

## Conclusions

The United Nations currently has limited ability to take on the problems of a globalized world and has limited capacity to affect major issues within member states. But it can have a useful influence in facilitating stronger oversight by broader civil society. It can do this by strengthening the national and global civil society voice in WHO and ILO structures, and by keeping conflict of interest out of policy decisions. Corporate influence on international organisations is not a new problem. It goes on in all member states and is evidenced in the neglect of occupational health and safety, and the weakness of workers’ compensation laws, in all developing countries.

UN agencies should develop stronger and unambiguous processes to manage conflict of interest in ways that equalize the influence of powerful interests with those of communities, Non-Governmental Organizations, Civil Society Organizations and Social Movements [36] More support should be given to protect the WHO from industry attacks and to help it increase its supply of information on occupational health and safety to developing countries, free of industry influence.

The WHO provides information, but insufficient support, for developing countries about prevention, worker engagement, and risks including occupational diseases, introduction of occupational health and safety standards, and increased regulation of hazards with effective enforcement. The ILO’s generation of national occupational health and safety profiles to monitor the state of play in developing countries and to help to plan improvements is useful. Its success will, however, again depend on sufficient resources and staff being available to ensure reported advances are real and not simply cosmetic.

The lack of unconditional funding for the WHO and ILO severely impedes the development of successful international programs. The budgets are spent on office staffs and publications that overstate the scope of their operations. The accomplishments claimed by the agencies are not apparent and not evidenced. However, some regional examples of good practice are identified which suggest possible ways forward. The UN could strengthen the national and global civil society voice in WHO and ILO structures, and, by keeping conflict of interest out of policy decisions, ensure greater freedom to operate without interference. The WHO could increase information and support for developing countries to aid prevention initiatives on occupational health and safety. This could complement the expansion of ILO work on standards, and hence possibly contribute globally to an increased and necessary regulation of hazards with effective enforcement.

The Office of the United Nations High Commissioner for Human Rights (OHCHR) also hosts Special Rapporteurs (SRs) whose role is to examine a specific human rights theme of whom two are particularly relevant for occupational health. The SR on the Right to Health noted in 2012 that occupational health is an integral component of the right to health and in his report made a set of recommendations that address the needs of vulnerable worker populations, place obligations on States for the formulation and implementation of occupational health policies and programs with strong participation of workers [3]. Further, the SR on toxics has argued human rights must be integrated into occupational safety and health discussions at the national and international levels. Such global analyses provide leverage for civil society and organised labor to strengthen protections for workers’ health at all levels [37].

Central to contributing to such a shift will be the ILO’s commitment to collective bargaining and workers’ rights to a safe and healthy workplace. The ILO does endorse collective bargaining and workers’ rights to a safe and healthy workplace, including regulation and enforcement, in line with the ILO Decent Work and other programs. It would not therefore be a major change of policy for the WHO and ILO to expand these activities with appropriate funding.

Locating human rights treaty commitments and ILO occupational health and safety provisions as prerequisites within Trade Treaties could also trigger a positive emphasis on occupational health and safety globally. There is a marked inequality between trade treaties and human rights treaties. The inequality can, however, be mitigated by UN agencies. Countries sign and ratify human rights treaties because there is no cost to them if they are flouted. Trade treaties, on the other hand, extract substantial economic punishments when violations are confirmed. If trade treaties were required to fully endorse human rights treaties in their enforcement mechanisms, they would bring about a dramatically different emphasis on occupational health and safety. If human rights treaties and ILO Conventions had more enforceability through trade treaties, some progress might well be made. Even if the change is slight, the impact is substantial.

The staff assigned to WHO and ILO agencies responsible for occupational health and safety should have appropriate credentials and backgrounds. The selection process is currently removed from public view, and not subject to approval by relevant international authorities. There is no current method of finding conflicts of interest in staff assignments. An international organization with no industry bias exists in the Collegium Ramazzini, headquartered in Bologna, Italy. The Collegium should be considered as an independent approval authority for WHO and ILO staff positions, and for technical review of publications.

## Abbreviations

CRI: Chulabhorn Research Institute
ECLT: Eliminating Child Labor in Tobacco
GCC: Gulf Cooperation Council
GNI: Gross National Income
HRW: Human Rights Watch
IARC: International Agency for Research on Cancer
ILO: International Labor Organization
MNE: Multinational Enterprises and Social Policy (MNE Declaration)
NGO: Non-Governmental Organization
PM: Philip Morris
SR: Special Rapporteur
UN: United Nations
USDG: Universal Sustainable Development Goals
WHO: World Health Organization
